# Are psychosocial stressors associated with the relationship of alcohol consumption and all-cause mortality?

**DOI:** 10.1186/1471-2458-14-312

**Published:** 2014-04-04

**Authors:** Esther Ruf, Jens Baumert, Christa Meisinger, Angela Döring, Karl-Heinz Ladwig

**Affiliations:** 1 Institute of Epidemiology II, Helmholtz Zentrum München, German Research Center for Environmental Health, Ingolstädter Landstr. 1, 85764 Neuherberg, Germany; 2 MONICA/KORA Myocardial Infarction Registry, Central Hospital Augsburg, Stenglinstr 2, 86156 Augsburg, Germany; 3 Institute of Epidemiology I, Helmholtz Zentrum München, German Research Center for Environmental Health, Ingolstädter Landstr. 1, 85764 Neuherberg, Germany; 4 Department of Psychosomatic Medicine and Psychotherapy, Klinikum rechts der Isar, Technische Universität München, Langerstr. 3, 81675 München, Germany

**Keywords:** Alcohol, Confounding, Mortality, Psychosocial stressors

## Abstract

**Background:**

Several studies have shown a protective association of moderate alcohol intake with mortality. However, it remains unclear whether this relationship could be due to misclassification confounding. As psychosocial stressors are among those factors that have not been sufficiently controlled for, we assessed whether they may confound the relationship between alcohol consumption and all-cause mortality.

**Methods:**

Three cross-sectional MONICA surveys (conducted 1984–1995) including 11,282 subjects aged 25–74 years were followed up within the framework of KORA (Cooperative Health Research in the Region of Augsburg), a population-based cohort, until 2002. The prevalences of diseases as well as of lifestyle, clinical and psychosocial variables were compared in different alcohol consumption categories. To assess all-cause mortality risks, hazard ratios (HRs) were estimated by Cox proportional hazards models which included lifestyle, clinical and psychosocial variables.

**Results:**

Diseases were more prevalent among non-drinkers than among drinkers: Moreover, non-drinkers showed a higher percentage of an unfavourable lifestyle and were more affected with psychosocial stressors at baseline. Multivariable-adjusted HRs for moderate alcohol consumption versus no consumption were 0.74 (95% confidence interval (CI): 0.58-0.94) in men and 0.87 (95% CI: 0.66-1.16) in women. In men, moderate drinkers had a significantly lower all-cause mortality risk than non-drinkers or heavy drinkers (p = 0.002) even after multivariable adjustment. In women, moderate alcohol consumption was not associated with lowered risk of death from all causes.

**Conclusions:**

The present study confirmed the impact of sick quitters on mortality risk, but failed to show that the association between alcohol consumption and mortality is confounded by psychosocial stressors.

## Background

A repeated observation in diverse populations is that light to moderate alcohol consumption provides a window of protection in which adverse health effects are outweighed by benefits. Numerous epidemiological studies reported J- or U-shaped curves when describing the association between levels of alcohol consumption and all-cause or cardiovascular mortality [1-4], showing an increased mortality risk among abstainers and heavy drinkers compared to light or moderate drinkers. These risk patterns have been shown to be less pronounced in women than in men [5].

Several pathophysiological mechanisms are considered to be responsible for the beneficial effect of moderate alcohol consumption, which increases serum HDL and lowers fibrinogen and tryglyceride concentrations as well as blood pressure [6]. In addition to the protective effect of moderate drinking derived from pathophysiological features of alcohol [7] and the inclusion of subjects who abstain because of illness (so-called ‘sick quitters’) in the non-drinking group [8], unmeasured confounders might be responsible for this alcohol-mortality relationship. Although there is evidence that shows beneficial trends in biomarker concentrations as a result of low to moderate alcohol intake [9], psychosocial stressors are among those factors that have not been sufficiently controlled for, indicating that the potential confounding of the relationship between alcohol and all-cause mortality by these stressors has not yet been clearly assessed.

Andreasson [10] suspects that J-shaped curves may be the result of complex associations between psychosocial stressors, other potential confounding factors and health conditions. There is broad evidence that psychosocial stressors are associated with increased cardiovascular and overall mortality, as shown for depression [11-13], work stress [14,15], impaired self-perceived health [16,17] and, less consistently, for anxiety [18,19]. Several cross-sectional studies have indicated that psychosocial variables have the same J- or U-shaped curves in relation to alcohol consumption as to mortality benefits [20-26]: non-drinkers and heavy drinkers alike may experience higher levels of depression and anxiety, suffer more frequent from bodily pain and perceive themselves to be less healthy, to have less vitality, and to be generally less sociable than moderate drinkers. Hence, the established J- or U-shaped relationship between alcohol consumption and mortality might be confounded by psychosocial stressors.

Daily living may exert numerous psychosocial stressors on the individual, including external stressors (e.g. work stress, low employment status, living alone), and internal stressors (e.g. depressive symptoms, intrusive impact of bodily symptoms). Minimizing emotional response to stress may lower psychological distress [23]. Higher levels of psychological distress are associated with the frequency and the volume of alcohol consumption [27]. Therefore, moderate alcohol consumption as a sign of a person’s favourable lifestyle may serve as a surrogate marker for a spectrum of positive mental health factors that attenuates the effect of chronic stressors on health [18]. This general positive health behaviour and the reduced exposure of psychosocial stressors may be most important as this is especially likely to reduce risk of illness.

While lifestyle and clinical risk factors have been considered in many of the studies investigating the association of alcohol consumption and mortality, there is a substantial lack of studies that controlled for psychosocial stressors. Therefore, the present study aims to set its focus to this class of potential confounders. Using data from the population-based MONICA/KORA Augsburg Cohort Study, we investigated if psychosocial stressors confound the relationship between alcohol consumption and all-cause mortality by comparing models with and without psychosocial stressors included.

## Methods

### Study design

The data of the present study were derived from the population-based MONICA (MONItoring trends and determinants in CArdiovascular disease) Augsburg Study as part of the multinational WHO MONICA project [28]. Altogether 13,427 subjects (6,725 men, 6,702 women, response 77%) aged 25–64 years (S1) and 25–74 years (S2, S3), respectively, randomly drawn from the general population with German nationality of the city of Augsburg and two adjunct counties (Southern Germany), participated in at least one of the three independent cross-sectional surveys, conducted in 1984/85 (S1), 1989/90 (S2) and 1994/95 (S3). All subjects were prospectively followed up within the framework of the Cooperative Health Research in the Region of Augsburg (KORA) until 2002 [29]. Mortality was ascertained by regularly checking the vital status of all sampled persons of the MONICA surveys through the population registries. In 1997–1998 and 2002–2003 the health status of all living persons was assessed using follow-up questionnaires. The MONICA surveys S1, S2 and S3 with the baseline examination were approved by the data protection commission following the rules at the time of the examinations (1984/85, 1989/90 and 1994/95). The follow-up examinations within the KORA framework were approved by the ethics committee of the Bavarian Medical Association. All studies were performed in accordance with the Declaration of Helsinki. All participants provided written informed consent.

### Study population

Among the MONICA/KORA sample of 13,427 subjects, 12,887 subjects (96.0%) had available psychosocial data. Of those, 3,917 (30.4%) were from S1, 4,539 (35.2%) from S2 and 4,431 (34.4%) from S3. The psychosocial data set extended the MONICA core design and followed recommendations given by the MONICA steering committee [30]. Eight participants had missing data in alcohol consumption and were excluded to assess the prevalence of diseases, leading to a total of 12,879 subjects.

For mortality analyses, participants suffering from disease conditions or undergoing treatments that require abstinence from alcohol (diabetes N = 508, heart failure N = 628), from severe diseases (myocardial infarction N = 255, cancer N = 142, only S1) or from diseases indicating possible former high alcohol consumption (liver disease N = 394) were excluded to avoid bias due to so-called ‘sick quitters’. A drop-out analysis revealed that disease burden was highest in the abstainer group, who had higher prevalences of a history of myocardial infarction, heart failure, diabetes and liver disease. No substantial differences with respect to drinking patterns were observed for participants with a history of cancer. A total of 1,599 participants (911 men and 688 women) suffering from at least one severe disease condition (which may influence drinking behaviour) were excluded leading to a study population of 11,282 participants (5,540 men and 5,742 women) for mortality analyses. For several variables, analyses were not possible within the whole study group because of missing data or because variables had not been investigated in all three surveys.

### Study endpoint

The study endpoint was all-cause mortality. Death certificates were obtained from the local health departments and were coded for the underlying cause of death using the ninth revision of the International Classification of Diseases (ICD 9) [31]. The duration of the follow-up was calculated as the interval between the baseline examination and death from all-causes, or the date of the last available follow-up information which was drawn from KORA follow-up examinations in 1997–1998 or 2002–2003 or from population registries. The last possible observation date was December 31, 2002. The cohort in the present analysis was followed for an average of 12.0 years (standard deviation 4.4) ranging from 0.1 to 18.2 years. During this observation period, 970 (15.0%) men and 479 (7.5%) women had died.

### Risk factor assessment at baseline

Baseline information on sociodemographic variables, alcohol consumption, smoking status, physical activity level, medical history and medication use was assessed by trained medical staff during a standardized interview. Additionally, all participants underwent an extensive standardized medical examination that included the collection of a non-fasting blood sample.

Psychosocial variables were answered by a self-administered questionnaire.

#### Alcohol consumption

In the standardized interview, alcohol intake was assessed by the following questions: ‘How much beer/wine/spirits did you drink over the previous weekend (Saturday and Sunday)?’, ‘How much beer/wine/spirits did you drink on the previous workday (or on the previous Thursday, if Friday was the previous workday)?’ Total intake was calculated by multiplying weekday consumption by five and adding this to weekend consumption, applying the following conversions: 1 liter beer = 40 g alcohol, 1 liter wine = 100 g alcohol, 1 shot distilled spirits (0.02 liter) = 6.2 g alcohol. Finally, the average number of grams of alcohol consumed per day (g/day) was derived. This 7-day recall method was validated against a 7-day diet record method in a subsample and revealed sufficient validity [1].

**Figure 1:**
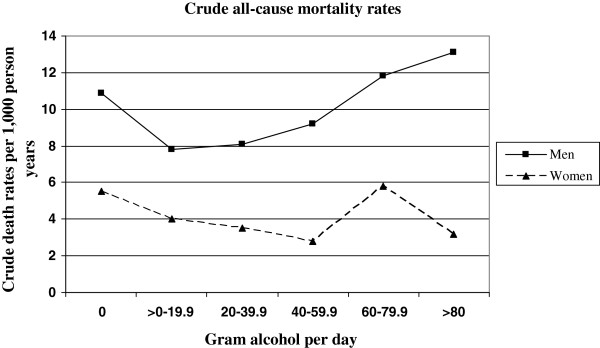
Crude all-cause mortality rates for alcohol consumption in men and women (n = 11,282 after exclusion of ill persons at baseline).

For the present analysis, alcohol consumption was classified into three categories: no alcohol consumption (0 g/day), moderate alcohol consumption (0.1-39.9 for men and 0.1-19.9 for women) and heavy alcohol consumption (≥ 40 g/day for men and ≥ 20 g/day for women) following previous studies regarding cardiovascular and all-cause mortality [1,32]. A broader classification into six categories was used when analysing crude death rates (see Figure 1), but was not used for the main analyses due to low case numbers and rather similar mortality risks in the three high alcohol consumption categories (40–59.9, 60.0-79.9, ≥ 80 g/day).

#### Lifestyle and clinical variables

Participants were classified as regular smokers when they reported that they currently smoke at least one cigarette per day.

To assess physical inactivity, participants were classified as ‘physically inactive’ during leisure time if they did not regularly participate in sports and if they were not active for at least 1 h per week in summer and winter.

Body height and body weight were determined by trained medical staff following a standardized protocol. Body mass index was calculated as weight in kilograms divided by high in square meters. According to the recommendations of the WHO, obesity was defined as BMI ≥ 30 kg/m^2^.

Blood pressure was measured using the right arm in a sitting position using a Hawksley random-zero sphygmomanometer adhering to the WHO MONICA protocol [29]. Actual hypertension was defined as blood pressure value ≥ 140/90 mmHg or use of antihypertensive medication, which indicates that the subject was aware of being hypertensive.

A non-fasting venous blood sample was collected from all participants while sitting. Total serum cholesterol (mg/dl) and high-density lipoprotein cholesterol (HDL-C) after precipitation with phosphotungstic acid/Mg2+, were measured by enzymatic methods (CHOD-PAP, Boehringer Mannheim, Germany). Dyslipidemia was defined as a ratio of total cholesterol to HDL-C ≥ 5.

Finally, history of diabetes, heart failure, myocardial infarction, cancer and liver diseases were assessed by self-reports of the participants and used for the definition and exclusion of so-called ‘sick quitters’.

#### Psychosocial stressors

Educational level was categorized into ‘low’ (≤ 12 years of schooling) and ‘high’ (> 12 years of schooling). Occupational status was classified into three categories: ‘currently employed’, ‘unemployed’ (i.e. subjects seeking work) and ‘not employed’ (i.e. homemakers or subjects in periods of education, parental leave or retirement). Social support was assessed according the Social Network Index (SNI) initially designed for the Alameda county study [33] comprising marital status, contact with friends and relatives, and an index of close contacts and activities in groups. The combination of all components allows a comprehensive rating from 1 (low SNI) to 4 (high SNI). SNI was classified into three categories using tertiles of the distribution (low, medium, high). Living alone was defined by marital status (living alone or being separated, divorced or widowed). Job strain symptoms, according to Karasek [34], were also classified into three categories using tertiles of the distribution (low, medium, high). Low educational level, unemployment, low social network, living alone, and high job strain were considered as *external stressors*.

Self-perceived health was directly assessed by the interview question: ‘How would you assess your current health condition - excellent, good, fair, poor?’ These four categories were dichotomized to an ‘excellent/good’- and a ‘fair/poor’-group [35]. Depressive symptoms were assessed using a subscale from the von Zerssen affective symptom check list [36,37]. Subjects in the upper third of the depressive symptom distribution were considered as the index group for subjects with a depressed mood. Severe somatic complaints were examined by nine items from the von Zerssen affective symptom check list [36]. The subscale combined nine items (e.g. sweating, palpitation, numbness, vertigo) ranging from 0 to 3, leading to a score range of 0–27. The internal consistency (measured by Cronbach’s α) of the somatic complaints subscale was high. The variable ‘level of somatic complaints’ was defined by using sex-specific tertiles of the distribution of the somatic complaints subscale (low, medium, high). Low self-perceived health, depressed mood and high level of somatic complaints were chosen as markers of *internal stressors*.

### Statistical analysis

Since the risk which is attributable to alcohol consumption differs by sex, all analyses were performed separately for men and women.

Crude incidence rates of all-cause mortality were estimated by the person-years method [38]. Cox proportional hazards models were calculated to assess the relative risk of all-cause mortality for moderate and high alcohol consumption compared to no alcohol consumption. The proportional hazards assumption was assessed by plotting the [−log(survival)] curves for each risk factor showing that proportional hazards could be assumed for all risk factors. Relative risks were computed as hazard ratios (HRs) with 95 percent confidence intervals (95% CI). The first ‘crude’ model included age (continuous) and survey (S1, S2 or S3), followed by the second ‘lifestyle/clinical’ model which included additionally the lifestyle variables regular smoking (yes or no), physical inactivity (yes or no), and obesity (yes or no) as well as the clinical variables actual hypertension (yes or no) and dyslipidemia (yes or no). The third ‘psychosocial stressors’ model included age, survey and additionally the psychosocial stressors low educational level (≤ or > 12 years), not employed (yes or no), low social network (yes or no), living alone (yes or no), high job strain (yes or no), low self- perceived health (yes or no), depressed mood (yes or no) and high level of somatic complaints (yes or no). The fourth ‘full’ model included age, survey and all lifestyle, clinical and psychosocial factors. Moreover, we tested possible interactions between alcohol consumption and all psychosocial factors on all-cause mortality risk.

Significance tests were 2-tailed. For all statistical analyses a P value less than 0.05 was considered to be statistically significant. The Akaike’s Information Criterion (AIC) was used to assess the goodness of fit of the models. The evaluations were performed with the statistical software package SAS (Version 8.02, SAS-Institute Inc., Cary, NC, USA).

## Results

### Descriptive analyses

In the study population (N = 11,282), a total of 850 (15.3%) males and 2,401 (41.8%) females reported no alcohol consumption. A total of 2,833 (51.1%) men and 2,230 (38.8%) women were classified to be moderate drinkers. Heavy alcohol consumption was observed in 1,857 (33.5%) men and 1,111 (19.3%) women.

Baseline characteristics stratified by alcohol consumption groups from the apparently healthy study sample (N = 11,282), adjusted for age and survey, are displayed in Table 1. The moderate consumption group reported significantly lower percentages in a number of lifestyle and clinical factors (e.g. physical inactivity, obesity, dyslipidemia) in comparison to heavy drinkers and non-drinkers. When considering psychosocial stressors, moderate drinkers reported significantly lower percentages of external stressors such as low educational level, low social network and living alone. Regarding internal stressors, no differences in depressive mood frequency was observed between the alcohol consumption groups in men. In women, moderate drinkers reported significantly less somatic complaints.

**Table 1:** Prevalence of medical and lifestyle variables and psychosocial stressors by alcohol consumption in men and women, adjusted for age and survey (n = 11,282 after exclusion of diseased participants at baseline)

	**Alcohol consumption (g/day)**							
	**Men**				**Women**			
	***(n = 5,540)***				***(n = 5,742)***			
	**0**	**> 0 – 39.9**	**≥ 40**	*p*-value*	**0**	**> 0 – 19.9**	**≥ 20**	*p*-value*
	**(*n = 850)***	***(n = 2,833)***	***(n = 1,857)***		***(n = 2,401)***	***(n = 2,230)***	***(n = 1,111)***	
Regular smoking [%]	34.4	28.0	40.7	<0.0001	20.0	22.2	26.8	<0.0001
Physically inactive [%]	56.6	51.5	56.1	0.0015	65.5	57.6	54.7	<0.0001
Obesity [%]	19.6	16.8	16.8	0.1222	23.6	16.2	12.1	<0.0001
Actual hypertension [%]	39.9	39.3	46.5	<0.0001	30.0	27.5	29.9	<0.0838
Dyslipidemia [%]	52.9	44.3	35.6	<0.0001	18.8	15.1	10.5	<0.0001
Low educational level [%]	76.3	71.4	78.2	<0.0001	85.5	82.8	77.6	<0.0001
Not employed [%]	28.7	25.6	19.8	<0.0001	55.6	51.7	47.7	<0.0001
Low social network^1^ [%]	53.8	48.2	45.6	0.0004	58.1	51.7	54.4	<0.0001
Living alone [%]	20.3	16.7	17.6	0.0457	23.9	23.2	24.7	0.5620
High job strain [%]	21.8	22.6	24.2	0.3049	16.9	18.0	17.7	0.5996
Low self-perceived health [%]	20.9	16.1	15.7	0.0016	26.9	20.5	18.9	<0.0001
Depressed mood^1^ [%]	34.9	35.4	33.1	0.2693	34.8	33.5	36.7	0.2069
High level of somatic compl.^1^ [%]	32.9	34.4	37.1	0.0570	34.2	29.5	30.3	<0.0018

*Wald *χ2-*Test.

^1^missings 5- < 10%.

Crude all-cause mortality rates for six alcohol consumption categories are displayed in Figure 1, indicating a J-shaped curve for men, but not for women. Crude rates of all-cause mortality per 1,000 person-years by three categories of alcohol consumption are presented in Table 2. In men, the crude rate of all-cause mortality was lower in moderate drinkers than in abstainers and heavy drinkers. In women, the crude rate of all-cause mortality was highest in abstainers, whereas no difference between moderate or heavy drinkers could be seen. Compared to women, men with no or moderate alcohol consumption had a twofold higher mortality rate and men with high alcohol consumption a threefold higher mortality rate, respectively.

**Table 2:** Age- and survey- and multivariable-adjusted hazard ratios and 95% confidence intervals for alcohol consumption in men and women (n = 11,282 after exclusion of ill persons at baseline)

	**Number of person-years**	**Number of deaths**	**Crude death rate/1,000 person-years**	**Model 1**	**Model 2**	**Model 3**	**Model 4**
				**Age- and survey- adjusted**	**Multivariable- adjusted**^**2**^	**Multivariable- adjusted**^**3**^	**Multivariable- adjusted**^**4**^
				**HR (95% CI)**^**1**^	**HR (95% CI)**	**HR (95% CI)**	**HR (95% CI)**
*Men (n=5,540)*							
1: 0 g/d	9,596	105/850	10.9	1.0	1.0	1.0	1.0
2: > 0–39.9 g/d	33,703	262/2,833	7.8	0.68 (0.54-0.85)	0.71 (0.56-0.90)	0.73 (0.57-0.93)	0.74 (0.58, 0.94)
3: ≥ 40 g/d	23,406	250/1,857	10.7	0.99 (0.79-1.24)	0.94 (0.75-1.20)	1.11 (0.87-1.42)	1.03 (0.80, 1.32)
*p-value*	*-*	*-*	*-*	*<0.0001*	*0.0010*	*<0.0001*	*0.0019*
*Women (n=5,742)*							
1: 0 g/d	28,722	159/2,401	5.5	1.0	1.0	1.0	1.0
2: > 0–19.9 g/d	27,790	106/2,230	3.8	0.80 (0.62-1.02)	0.87 (0.68-1.12)	0.81 (0.61-1.07)	0.87 (0.66, 1.16)
3: ≥ 20 g/d	14,306	55/1,111	3.8	0.87 (0.64-1.18)	0.92 (0.67-1.26)	0.86 (0.60-1.22)	0.90 (0.63, 1.29)
*p-value*	*-*	*-*	*-*	*0.1886*	*0.5541*	*0.3082*	*0.6119*

^1^HR hazard ratio; CI confidence interval.

^2^‘Lifestyle/clinical model’: Adjusted for age, survey, regular smoking, physical inactivity, obesity, actual hypertension and dyslipidemia.

^3^‘Psychosocial stressors model’: Adjusted for age, survey, low educational level, not employed, low social network, living alone, high job strain, low self- perceived health, depressed mood and high level of somatic complaints.

^4^‘Full model’: Adjusted for age, survey, regular smoking, physical inactivity, obesity, actual hypertension, dyslipidemia, low educational level, not employed, low social network, living alone, high job strain, low self- perceived health, depressed mood and high level of somatic complaints.

### Cox regression analyses

The all-cause mortality risks estimated by four Cox proportional hazards models are given in Table 2 showing different results for men and women. For males, moderate alcohol consumers had a significantly reduced risk of mortality from all-causes compared to non-drinkers (HR: 0.68; 95% CI: 0.54-0.85, p < 0.0001) in a ‘crude’ model (adjusted for age and survey). This association remained stable after adjusting additionally for lifestyle and clinical factors (HR = 0.71, 95% CI: 0.56-0.90, p = 0.001) or for psychosocial stressors (HR = 0.73, 95% CI: 0.57-0.93, p < 0.0001). In the ‘full’ model (adjusted for age, survey, lifestyle, clinical and psychosocial variables), male moderate drinkers still had a significantly reduced mortality risk with a comparable effect (HR: 0.74; 95% CI: 0.58-0.94, p = 0.002). In females, moderate drinkers had no significantly reduced risk of all-cause mortality compared to non-drinkers (HR = 0.80, 95% CI: 0.62-1.02, p = 0.189). The multivariable adjustment did not affect the relationship between alcohol consumption and mortality in women (see Table 2).

### Sensitivity and interaction analyses

We computed the same models as above with linear variables instead of dichotomized variables as the categorizations of the lifestyle, clinical and psychosocial variables might be too crude. However, we found comparable results. Additional models have been estimated to control for potential differential effects of psychosocial stressors on alcohol consumption by adding interaction terms between alcohol consumption groups and all psychosocial stressors. In men, a significant interaction was found between alcohol consumption and living alone (p = 0.030): In a stratified analysis, moderate alcohol consumers who lived alone had a reduced risk of all-cause mortality (HR = 0.48; 95% CI 0.28-0.82, p = 0.030), whereas subjects not living alone had a HR of 0.85 (95% CI: 0.64-1.13). In women, no significant interactions were found between alcohol consumption and psychosocial stressors with respect to all-cause mortality.

## Discussion

### Overall

The present investigation based on a large sample of apparently healthy subjects drawn from the general population confirmed that all-cause mortality is lowest in moderate drinkers, even after controlling for lifestyle and clinical variables as well as for a broad range of psychosocial stressors. Therefore, our data contradict the previously expressed assumption that there is ‘probably no free lunch’ [39] with drinking alcoholic beverages. To avoid the “sick quitters bias” we excluded subjects reporting disease at baseline as it is possible that these participants quit drinking because of adverse health experiences and therefore could be misclassified. Nonetheless, this exclusion may lead to reduced external validity because the study population does not fully represent the underlying general population anymore.

### Lifetime abstainers and ex-drinkers

The criticism that abstainers per se are a rather heterogeneous group and therefore not an appropriate comparison group is justified since “abstainers” may be lifetime abstainers or ex-drinkers [40]. Because health concerns are frequently related to having given up drinking, it has been argued that a separation of abstainers into lifetime abstainers and ex-drinkers leads to less pronounced or a complete disappearance of beneficial effects [8]. This hypothesis has been attenuated by findings which confirmed a protective association for moderate alcohol consumption and cardiovascular diseases even after separating recent abstainers from lifetime abstainers [41]. A large meta-analysis revealed that ex-drinkers had a higher mortality risk compared to lifetime abstainers. For women, this effect was less pronounced than for men [42]. In our study, after excluding participants reporting severe disease conditions at baseline from all analyses to avoid bias from sick quitters, we still found a J-shaped curve between alcohol consumption and all-cause mortality in men. In women, the J-shaped curve was not approximated, most likely due to a power problem resulting from very small numbers of women in the higher consumption groups. However, there might be several differences between lifetime abstainers and ex-drinkers which we were not able to account for such as previous problematic drinking patterns among ex-drinkers. Similar risks for negative health conditions were shown for former heavy drinkers compared to current heavy drinkers [43].

### Lifestyle factors

The present investigation confirmed earlier findings that male moderate drinkers have a positive lifestyle behaviour that favours their survival over non-drinkers (e.g. as physical activity and non-smoking behaviour) [44-49]. The benefits of moderate drinking relative to abstinence were present primarily within the context of an otherwise healthy behavioural profile [50].

### Psychosocial stressors

The hypothesis of the modification of the risk curve between alcohol and all-cause mortality by social isolation [51,52] has been empirically tested, but a direct protective effect of social integration has not been confirmed [53,54]. In our data, we found that the effect of alcohol consumption on all-cause mortality was modified by living alone or not alone in men: Whereas men who lived alone had a significantly reduced mortality risk compared to men reporting no alcohol consumption. No significant differences in mortality risk were found for the three alcohol consumption groups in men living not alone. Therefore, alcohol consumption had no effect on mortality in men living not alone. One explanation for this finding might be that men who did not live alone already had a reduced mortality risk compared to men living alone and therefore, there was no space for a significant decreased mortality risk by moderate alcohol consumption (however, a tendency toward risk reduction was found with HR 0.85).

There is evidence from several cross-sectional studies showing that non-drinkers as well as heavy drinkers experience higher levels of depression, psychological distress, anxiety and lower levels of subjective health than moderate drinkers [20-26,55]. In our study, there is no clear evidence of a generally lower level of psychosocial stressors in moderate drinkers in comparison to non-drinkers and heavy drinkers. Moderate drinkers reported somatic complaints less frequently, however, for depressed mood, no differences between the three consumption categories were found. The lack of significant differences in depressed mood and other measures of psychological distress in our study as opposed to other studies remains unclear; the use of different instruments might contribute to these inconsistencies. However, the present investigation did not provide compelling evidence that the J- or U-shaped relationship between alcohol and all-cause mortality could be explained by confounding from psychosocial stressors.

### Potential pathophysiological mechanism

Several pathophysiological potential mechanisms for the protective effect of moderate alcohol intake have been suggested [7]. The J-shape of mortality risk has been attributed to a combination of beneficial and harmful effects of ethanol itself. Among them are lower levels of inflammatory markers, improved flow-mediated vasodilation and favourable effects on serum lipid levels for subjects having a low to moderate alcohol intake compared to abstainers [56].

In addition to overall level of alcohol consumption, specific drinking patterns, especially heavy or so-called binge drinking, has also been shown to affect mortality; meta-analyses revealed lower cardiovascular risks as a result of regular daily intake of a low to moderate dose of alcohol, and a higher risk from infrequent binge drinking [57-59]. Overall, the causes of the advantageous health status of moderate drinkers must be regarded as multiple and should be further investigated.

### Strengths and limitations

The strengths of the present study are the prospective design, the large sample size based on a random sample drawn from the general population and the availability of a large set of lifestyle and clinical factors as well as numerous external and internal psychosocial stressors. Additionally, all factors were scrutinized by standardized and quality-controlled assessments. The large sample size allows a broad controlling for potential confounders. Excluding subjects already diseased at baseline from the analyses avoided misattribution of psychosocial variables to abstinence, as diseases could both lead to abstention and development of mood disorders or impaired social conditions.

The MONICA/KORA Augsburg Cohort Study has several limitations that need to be considered. Because the study was limited to men and women of German nationality, caution should be used in generalizing these results to people of other ethnicities. A general limitation of studies on alcohol is that self-reported alcohol intake is particularly susceptible to underreporting [60], not only due to selection and recall bias but also due to a tendency to give socially desirable answers. However, comparing the proportion of persons with elevated serum gamma-glutamyltransferase in the three alcohol consumption groups in S1 did not indicate a misattribution of drinkers in the abstinent group (data not shown). In addition, we couldn’t perform a multiple measurement of alcohol intake but only a single week and only a single day/weekend measurement. Therefore, only volume could have been analysed and not specific drinking patterns such as heavy drinking episodes. Furthermore, a major shortcoming is the fact that no differentiation between lifetime abstainers and current abstainers could be made. Another limitation that needs to be addressed is that depressive symptoms were measured using a symptom rating scale which is among the less rigorous options to assess depressive mood, although a recent re-examination of its validity and reliability is promising [37]. The results do not pertain to major depression as defined in international classification systems. Additionally, depressive symptoms were measured at one time point, so that transient states of depression could not be distinguished from persistent states.

## Conclusion

The present study analysed the impact of psychosocial stressors on the association of alcohol and all-cause mortality after excluding potential ‘sick quitters’. In men, moderate drinkers were at significantly lower all-cause mortality risk than non-drinkers or heavy drinkers. This pattern persisted after adjustment for lifestyle risk factors and clinical as well as for external and internal psychosocial stressors. In women, no protective effect of moderate drinking was shown. The observed protective effect of moderate drinking could not be attributed to misclassification or confounding by psychosocial stressors in a large cohort of men and women drawn from the general population in Southern Germany.

## Competing interests

The authors declare that they have no competing interests.

## Authors’ contributions

ER prepared the data, performed statistical analyses of the data, contributed to the interpretation of the findings and drafted the paper. JB performed statistical analyses of the data, contributed to the interpretation of the results and writing of the manuscript. CM and AD contributed to the interpretation of the findings. KHL had the idea of the study, supervised the study and contributed to the interpretation of the findings. All authors read and approved the final draft of the paper.

## Pre-publication history

The pre-publication history for this paper can be accessed here:

http://www.biomedcentral.com/1471-2458/14/312/prepub
